# Supplementary material for: Causes and consequences of sex-chromosome turnovers in Diptera
Source: Evol Lett. 2026 Mar 12;10(3):253–68. doi: 10.1093/evlett/qrag003 (PMC13231274; doi:10.1093/evlett/qrag003)
Supplement: qrag003_Supplemental_Files [file qrag003_supplemental_files.zip › Supplementary_Datasets.zip!/Supplementary_Datasets/Dataset_2_Graphical_representation_outgroups/Stratiomydae_Beris_morrisii_chromosomes.pdf]

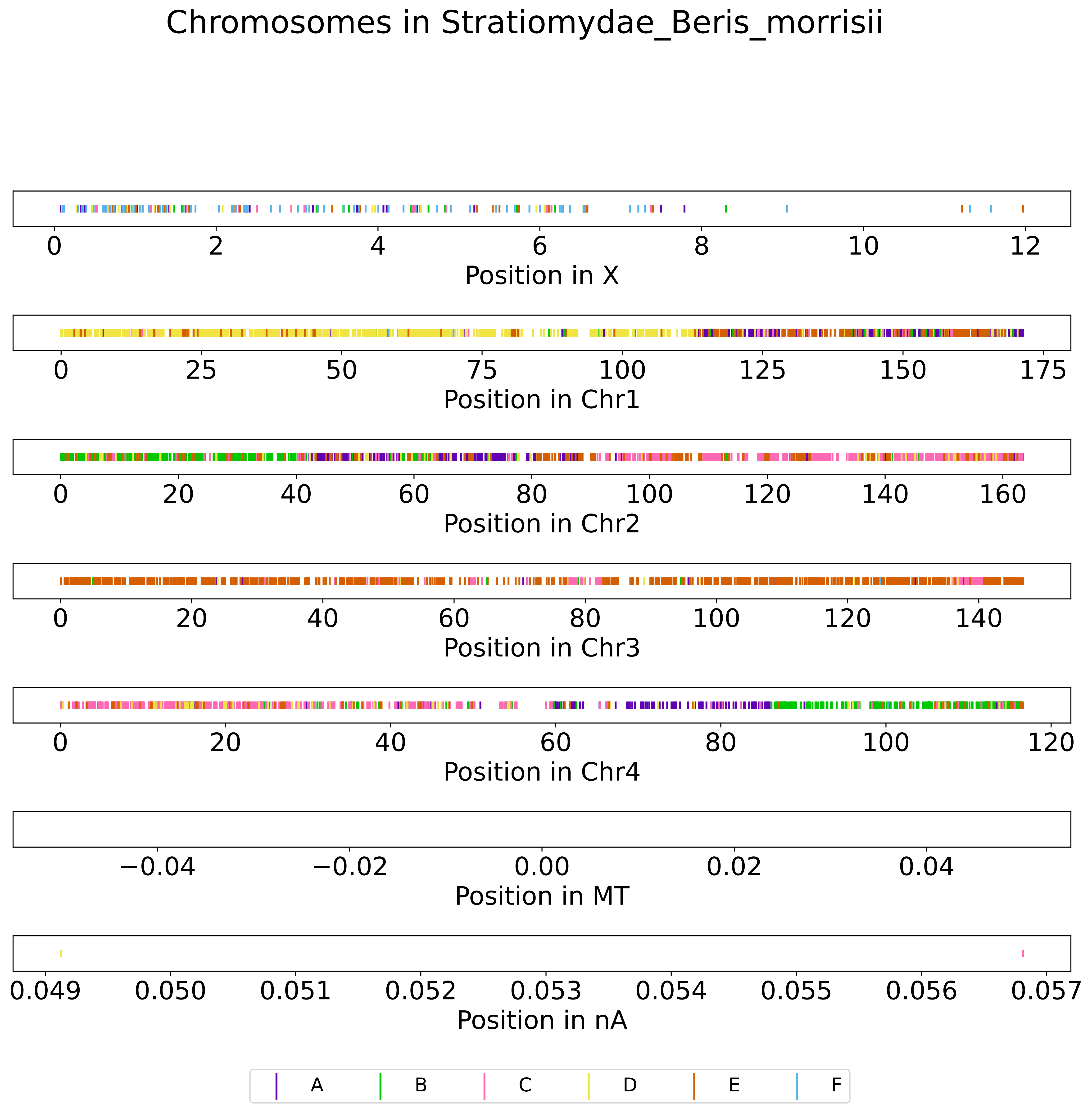

Chromosomes in Stratiomydae_Beris_morrisii
0 2 4 6 8 10 12
Position in X
0 25 50 75 100 125 150 175
Position in Chr1
0 20 40 60 80 100 120 140 160
Position in Chr2
0 20 40 60 80 100 120 140
Position in Chr3
0 20 40 60 80 100 120
Position in Chr4
−0.04 −0.02 0.00 0.02 0.04
Position in MT
0.049 0.050 0.051 0.052 0.053 0.054 0.055 0.056 0.057
Position in nA
A
B
C
D
E
F
